# Supplementary material for: Drug repurposing for aging research using model organisms
Source: Aging Cell. 2017 Jun 16;16(5):1006–15. doi: 10.1111/acel.12626 (PMC5595691; doi:10.1111/acel.12626)
Supplement: Supplementary file 7 — Data S1 Zip‐Archive of all report cards. [file ACEL-16-1006-s007.zip › RC_NCA.pdf]

NCA

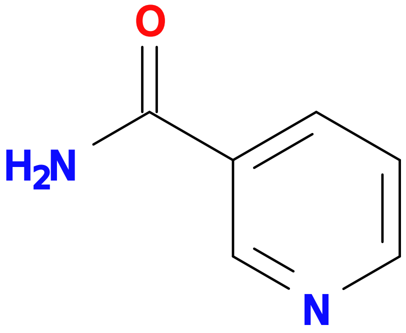

Database identifiers

ChEMBLCompound	CHEMBL1140
DrugBank	DB02701
CHEBI	17154
ZINC	ZINC00005878
eMolecules	27678415
eMolecules	490874

Ranking

	Rank	Score
Drosophila	369/697	0.403
C. elegans	115/591	0.309

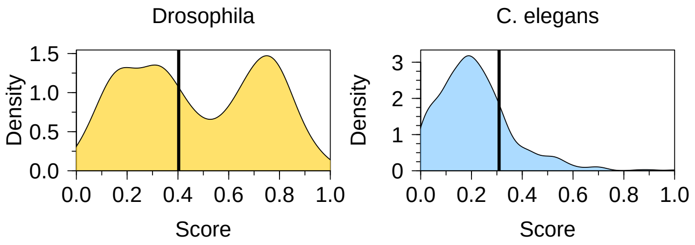

	Ageing implication	Domain conservation	Binding site conservation	Binding affinity	Bioavailability	Lipinski	Promiscuity	Purchasability	Drug approval	**Total**
Drosophila	1.0	0.897	0.795	0.317	(0.9)	0.0	-0.0	0.1	0.1	0.403
C. elegans	1.0	0.877	0.795	0.317	0.495	0.0	-0.0	0.1	0.1	0.309

Names

- **nicotinamide**
- 3-pyridinecarboxamide
- beta-pyridinecarboxamide
- niacin
- Niacinamide
- Nicotinic Acid Amide
- Nicotylamide
- Nikotinsaeureamid
- vitamin B3

Roles

metabolite, neuroprotective agent, EC 3.5.1.98 (histone deacetylase) inhibitor, anti-inflammatory agent, Sir2 inhibitor, EC 2.4.2.30 (NAD(+) ADP-ribosyltransferase) inhibitor, cofactor, antioxidant

Status

Approved drug (according to ChEMBL)	Yes
Number of Rule of 5 violations	0
Binding affinity to original target in log units (RF-Score prediction)	4.23
Burns *C. elegans* bioavailability prediction	2.79

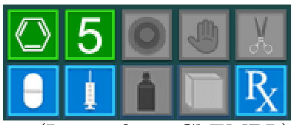

(Image from ChEMBL)

Compound Target Characteristics

NAD-dependent histone deacetylase SIR2

Best gene implication in ageing for this target family came from gene Q9VK34 annotated in UniProt release 2014_02. Annotation GO 8340 (determination of adult lifespan) was Inferred from Mutant Phenotype

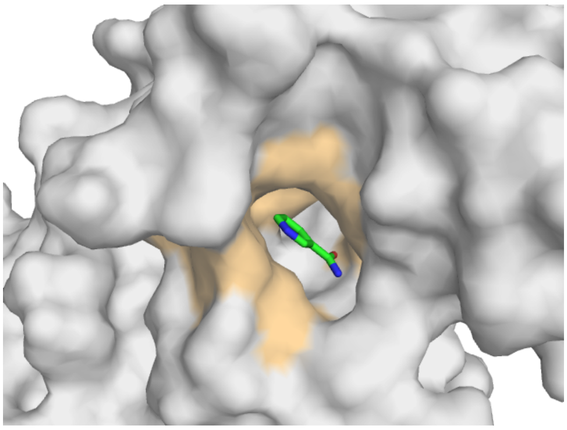

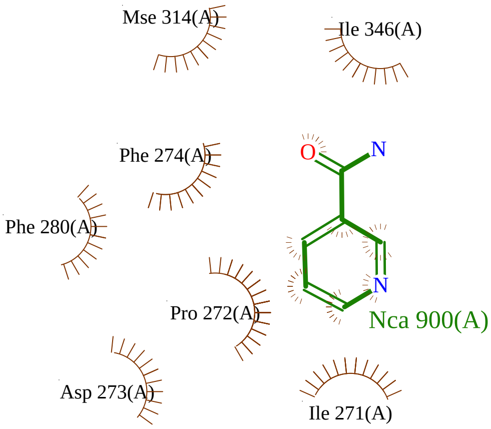

protein	amino acids contacts (binding site)							
PDB:2hjh:chainA:P06700	I	P	D	F	F	M	V	I
tr:A8K128:A8K128_HUMAN	I	P	D	F	I	E	I	I
sp:Q96EB6:SIR1_HUMAN	I	P	D	F	I	E	I	I
tr:F1M3P2:F1M3P2_RAT	I	P	D	F	I	E	I	I
tr:F1LTP2:F1LTP2_RAT	I	P	D	F	I	E	I	I
tr:Q3UNI1:Q3UNI1_MOUSE	I	P	D	F	I	E	I	I
tr:Q53Z05:Q53Z05_MOUSE	I	P	D	F	I	E	I	I
sp:Q923E4:SIR1_MOUSE	I	P	D	F	I	E	I	I
tr:Q86LE9:Q86LE9_DROME	I	P	D	F	I	E	I	I
sp:Q9VK34:SIR2_DROME	I	P	D	F	I	E	I	I
tr:I1V510:I1V510_DROME	I	P	D	F	I	E	I	I
tr:D3YT50:D3YT50_CAEEL	I	P	D	F	I	E	I	I
sp:Q21921:SIR2_CAEEL	I	P	D	F	I	E	I	I
sp:P53685:HST1_YEAST	I	P	D	F	F	M	V	I
sp:P06700:SIR2_YEAST	I	P	D	F	F	M	V	I

protein	whole protein ident	simil	domain-based ident	simil	contact-based ident	simil
PDB:2hjh:chainA:P06700	1.0	1.0	1.0	1.0	1.0	1.0
tr:A8K128:A8K128_HUMAN	0.16	0.31	0.45	0.8	0.63	0.8
sp:Q96EB6:SIR1_HUMAN	0.16	0.39	0.45	0.8	0.63	0.8
tr:F1M3P2:F1M3P2_RAT	0.16	0.32	0.45	0.8	0.63	0.8
tr:F1LTP2:F1LTP2_RAT	0.15	0.3	0.45	0.8	0.63	0.8
tr:Q3UNI1:Q3UNI1_MOUSE	0.17	0.39	0.45	0.8	0.63	0.8
tr:Q53Z05:Q53Z05_MOUSE	0.16	0.38	0.45	0.8	0.63	0.8
sp:Q923E4:SIR1_MOUSE	0.16	0.38	0.45	0.8	0.63	0.8
tr:Q86LE9:Q86LE9_DROME	0.23	0.51	0.49	0.82	0.63	0.8
sp:Q9VK34:SIR2_DROME	0.16	0.37	0.49	0.82	0.63	0.8
tr:I1V510:I1V510_DROME	0.16	0.37	0.49	0.82	0.63	0.8
tr:D3YT50:D3YT50_CAEEL	0.15	0.34	0.42	0.8	0.63	0.8
sp:Q21921:SIR2_CAEEL	0.16	0.36	0.42	0.8	0.63	0.8
sp:P53685:HST1_YEAST	0.55	0.77	0.85	0.96	1.0	1.0
sp:P06700:SIR2_YEAST	1.0	1.0	1.0	1.0	1.0	1.0

Sir2 (FBgn0024291) associated phenotypes

RU486 conditional, chemical resistant, conditional, dominant, enhancer of variegation, feeding behavior defective, hypoactive, increased cell death, long lived, male limited, non-suppressor of variegation, partially lethal - majority die, short lived, some die during pupal stage, suppressor of variegation

(Information from FlyBase)

Sir2 (UniProt:Q9VK34) annotation

Function: NAD-dependent histone deacetylase involved in heterochromatic silencing. Mildly suppresses the heterochromatin- mediated silencing phenomenon known as position-effect variegation (PEV). Required for epigenetic silencing of the polycomb group proteins. Has histone H4 deacetylase activity in vitro. Required maternally for establishing proper segmentation of the embryo. Involved in sex determination. May be involved in the regulation of life span. (PubMed:11281647, PubMed:12086602, PubMed:12663533, PubMed:15498488, PubMed:15520384, PubMed:17159295).
Cofactor: Zn(2+)Note=Binds 1 zinc ion per subunit. ;
Biophysicochemical properties: pH dependence: Optimum pH is 9. (PubMed:11281647);
Subunit: Interacts with the transcriptional repressors hairy (h) and deadpan (dpn); via basic domains. Associates with the Esc/E(z) histone methyltransferase complex. Interacts directly with E(z) and Rpd3. (PubMed:12086602, PubMed:15498488).
Subcellular location: Cytoplasm. Nucleus.
Disruption phenotype: Causes lethality during development. Induced silencing shortens life span. (PubMed:17159295).

(Information from UniProt)

sir-2.1 (WBGene00004800) associated phenotypes

drug response variant, fat content increased, organism oxidative stress response variant, protein expression increased, shortened life span

(Information from WormBase)

sir-2.1 (UniProt:Q21921) annotation

Function: NAD-dependent deacetylase (By similarity). Functions upstream of daf-16 in the insulin-like signaling pathway, promoting daf-16 mediated transcriptional activation and increased life-span. May also regulate life-span independently of daf-16 by modulating the transcription of genes involved in the stress response of the endoplasmic reticulum (ER). (, PubMed:11242085, PubMed:15793589, PubMed:16256736, PubMed:16280150, PubMed:16777605).
Cofactor: Zn(2+)Note=Binds 1 zinc ion per subunit. ;
Subunit: Interacts with ftt-2 and par-5. Interacts with daf-16 following heat-shock, which causes daf-16 to accumulate in the nucleus. Interaction with daf-16 is promoted by ftt-2. (PubMed:16777605).
Subcellular location: Nucleus ECO:0000269—PubMed:16280150, PubMed:16777605).

Developmental stage: Expressed in neurons of the head and tail from embryo to adult. Expressed in the hypodermis from the three- fold stage of embryogenesis; expression in the hypodermis subsequently decreases at L3 and is undetectable in adults. (PubMed:16280150).

(Information from UniProt)
